# Which Systematic Review Software Works Best? A Practical Comparison

**DOI:** 10.5195/jmla.2026.2262

**Published:** 2026-01-01

**Authors:** Juan José Serrano Silva, Sandra Fernández, Nicolás Rosillo, Héctor Bueno

**Affiliations:** 1 juanjo.serrano.silva@gmail.com, Cardiology Department, Hospital Universitario de Jerez de la Frontera, Jerez de la Frontera, Spain; Centro Nacional de Investigaciones Cardiovasculares (CNIC), Madrid, Spain; 2 sandra.fernandez@cnic.es, Centro Nacional de Investigaciones Cardiovasculares (CNIC), Madrid, Spain; Instituto de Investigación Sanitaria Hospital 12 de Octubre (i+12), Madrid, Spain; 3 nicorora93@gmail.com, Cardiology Department, Hospital Universitario 12 de Octubre, Madrid, Spain; Instituto de Investigación Sanitaria Hospital 12 de Octubre (i+12), Madrid, Spain; 4 hbueno@cnic.es, Centro Nacional de Investigaciones Cardiovasculares (CNIC), Madrid, Spain; Cardiology Department. Hospital Universitario 12 de Octubre, Madrid, Spain; Instituto de Investigación Sanitaria Hospital 12 de Octubre (i+12), Madrid, Spain

## Abstract

**Covidence**. Covidence Pty Ltd, Level 10, 446 Collins ST, Melbourne VIC 3000, Australia; support@covidence.org; https://www.covidence.org/; pay per review.

**Rayyan**. Rayyan, 1 Broadway, 14th Floor Cambridge, MA, 02142 USA; https://www.rayyan.ai/; pay per user.

**EPPI Centre**. EPPI Centre, Social Science Research Unit, UCL Social Research Institute, 10 Woburn Square, London WC1H 0NS; eppisupport@ucl.ac.uk; https://eppi.ioe.ac.uk/cms/; pay per user.

**Distiller SR**. DistillerSR Inc, 505 March Road, Suite 450, Ottawa, Ontario, Canada, K2K 3A4; support@distillersr.com; https://www.distillersr.com/; contact for pricing.

**RevMan**. The Cochrane Collaboration, 11-13 Cavendish Square, London, W1G 0AN, United Kingdom; https://revman.cochrane.org/info; pay per user.

Systematic reviews are critical in evidence-based medicine, yet their execution demands substantial resources in both time and personnel. The growing volume of scientific publications, the adoption of increasingly rigorous methodological standards, such as PRISMA [1,2], the use of evidence-quality assessment tools [3] and the need of conducting exhaustive searches across multiple databases [4] have amplified their complexity and workload. This complexity underscores the need for specialized tools to optimize the review process. This analysis summarizes and compares the leading software for systematic reviews and meta-analyses, showing how an informed choice can enhance both efficiency and quality. To this end, we conducted a targeted literature review of the most commonly used software for systematic reviews and meta-analyses followed by a critical evaluation of their features to guide researchers in selecting the tool best suited to their needs.

The most widely used softwares for conducting systematic reviews are Covidence [5], Rayyan [6], EPPI-Reviewer [7], DistillerSR [8], and Review Manager (RevMan) [9].

Covidence is widely recognized for its intuitive interface—usually associated with a shorter learning curve— and its capacity to streamline screening and data extraction. As a web-based platform, it facilitates real time collaboration among team members. Its pricing model is based on a per-review fee, allowing unlimited users per project, an advantage for teams with many contributors. Rayyan, in contrast, offers a basic free version (with optional paid) and leverages artificial intelligence to accelerate screening and duplicate detection. It is particularly accessible and integrates well with reference managers. Its paid model is user-based, making it potentially more cost-effective for smaller teams. However, Rayyan lacks built-in functionalities for data extraction and quality assessment, which limits its utility beyond the initial screening phases. Despite these limitations, both Covidence and Rayyan are excellent, low-cost solutions for researchers prioritizing efficiency and collaboration in the early stages of a systematic review. Neither, however, offers meta-analysis capabilities.

For more advanced requirements, platforms such as EPPI-Reviewer or DistillerSR provide extended functionalities, including machine learning tools and comprehensive process automation. These solutions offer greater power and flexibility but are generally more complex, with steeper learning curves and higher costs. Their ability to integrate with other systems and workflows varies by platform. RevMan, the reference software supported by the Cochrane Foundation, stands out for its user-friendly environment for data analysis and writing. Although it lacks automation capabilities and robust screening functionalities, it includes built-in meta-analysis functions and generates standard graphs such as forest plots. Its limited interoperability with external applications, however, may be a constraint in more integrated or customized workflows. A detailed comparison of the features, strengths, and limitations of these platforms is provided in Table 1.

**Table 1:**
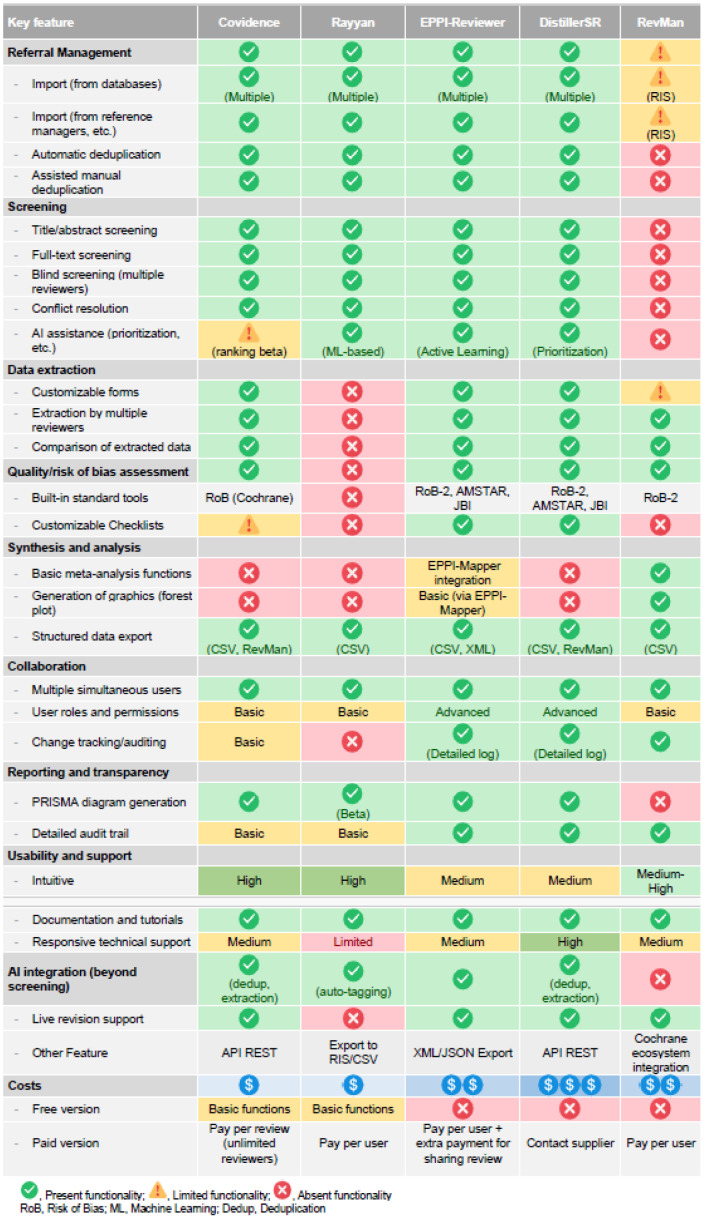
A comparative analysis of the features, strengths, and limitations of leading software for systematic reviews.

While it is theoretically possible to conduct a systematic review without dedicated software, doing so is inefficient, time-consuming, and increases the risk of error. Critical stages such as duplicate removal, study screening and quality assessment, data extraction, and collaborative analysis can benefit substantially from the use of specialized tools. The selection of a specific platform depends on multiple factors, including the complexity of the review, team size, budget constraints, required functionalities, acceptable learning curve, and compatibility with the researcher's existing digital ecosystem.

Importantly, the choice of software does not have to be limited to a single tool. An optimal workflow may involve the combined use of several platforms—such as employing Rayyan for its efficient screening capabilities, followed by export to RevMan for meta-analysis and reporting. Therefore, prioritizing and tailoring tool selection to specific needs of each phase is essential. Ultimately, the strategic use of appropriate software is critical to enhancing the efficiency of research teams and ensuring the methodological rigor and overall quality of systematic reviews.
